# Substandard medicines in Nepal: a crisis of access, equity, and a call for action

**DOI:** 10.3389/fpubh.2025.1584872

**Published:** 2025-08-29

**Authors:** Animesh Ghimire

**Affiliations:** ^1^Sustainable Prosperity Initiative Nepal, Kathmandu, Nepal; ^2^Faculty of Medicine, Nursing and Health Science, Monash University, Melbourne, VIC, Australia

**Keywords:** drug safety, Nepal, pharmaceutical quality, health equity, universal health coverage

## Introduction

Substandard medicines in Nepal represent an under-documented yet critical threat to universal health coverage (UHC) and patient safety, disproportionately impacting marginalized communities. This opinion piece seeks to illuminate the prevalence, drivers, and far-reaching consequences of compromised pharmaceuticals, drawing on both local and global evidence. This paper offers pragmatic strategies to strengthen regulatory capacity and accountability by spotlighting ethical imperatives and systemic gaps. Bridging best practices from international frameworks with Nepal's unique context, I aim to catalyze policy discourse, cultivate public awareness, and spur multi-sectoral collaboration. Ultimately, the goal is to foster equitable and high-quality healthcare for all, reinforcing the moral imperative of safeguarding the integrity of Nepal's medicine supply.

## A moral imperative: eradicating substandard medicines in Nepal

Substandard medicines are a pressing, under-recognized public health crisis in Nepal. Despite expanded healthcare access, compromised pharmaceuticals undermine patient wellbeing and public trust (1, 2). The World Health Organization (WHO) estimates that at least one in 10 medicines in low- and middle-income countries (LMICs) is substandard or falsified (3). In Africa, reports indicate up to 18.7% of sampled pharmaceuticals are compromised (4). This global issue is significantly influenced by two of the world's major suppliers of medicine, both domestically and internationally: India and China (5). According to estimates from India's Health Ministry, 5% of the nation's medicines are counterfeit and 0.3% are spurious, with one study suggesting that 75% of counterfeit medicines supplied globally originate in India (6). While recent, precise prevalence data for counterfeit drugs from China is not readily available, its role as a global manufacturing powerhouse is undisputed; for example, the country produces ~120 billion aspirin tablets annually for the North American market alone (7). A 2024 literature review highlights the historical scale of the issue, noting that 31% of the world's falsified and counterfeit medications are produced in China and Hong Kong (8). This geopolitical context is critically important for Nepal. The country's porous borders with these major pharmaceutical producers (9), combined with its own under-regulated supply chains, significantly heighten its susceptibility to the circulation of substandard drugs (10).

A Nepal Health Research Council (NHRC) study found that 15.16% of 244 tested medicine batches failed pharmacopeial standards (11). Alarmingly, 62.16% of these were government-supplied. Compromised products included essential antibiotics, analgesics, and iron supplements, with failures in dissolution, assay, fill volume, and sterility. A 2010–2020 review of drug recall notices in Nepal revealed a surge in recalled products, most frequently antimicrobials (2). These findings indicate an ethical breach: public health facilities primarily serve the underprivileged, disproportionately imposing the burden of substandard medicines on them—a direct affront to the principle of justice (12). Patients, often unaware of compromised drug quality due to identical appearance, are denied meaningful informed consent. This erodes trust in the healthcare system and fuels antimicrobial resistance, a global health security threat with severe implications (3).

Limited regulatory capacity exacerbates these dangers. In the Nepalese context, chronic under-resourcing of the Department of Drug Administration (DDA) and the National Medicines Laboratory hampers rapid testing and enforcement (13, 14). Testing delays can extend for months, allowing compromised medications to circulate (15). While decentralizing drug procurement to local governments was intended to improve responsiveness, this policy has inadvertently weakened quality control due to a lack of local testing facilities and specialized personnel (14).

## Discussion

Nepal's crisis of substandard medicines undermines human rights and core ethical principles. Tolerating this compromise of patient safety is incompatible with universal health coverage (UHC) (16). In Nepal, where a significant portion of the population relies on public health facilities for essential medicines, the prevalence of substandard drugs, especially in public health facilities (17), directly undermines the core tenets of UHC. Specifically, it violates the principle of access to quality healthcare: even if services are nominally affordable or free, receiving ineffective or harmful medication means that true, effective healthcare is not being delivered. Furthermore, the consequences of substandard medicines—prolonged illness, treatment failure, or adverse drug reactions—often lead to increased financial hardship for patients who may require further treatment, hospitalization, or experience loss of income due to illness. This directly contradicts the UHC goal of protecting people from financial risks associated with healthcare (18). This creates a two-tiered system, where those with resources can seek higher-quality care in the private sector, while the most vulnerable are left exposed to potentially dangerous treatments. Addressing this inequity and ensuring the integrity of the medicine supply is not just a matter of policy but a fundamental requirement for social justice. Success requires sustained political commitment, collaboration across sectors, and a steadfast focus on the public interest. Nepal must act decisively to eliminate substandard medicines and ensure equitable, high-quality healthcare for every citizen. This is not merely a matter of improving healthcare statistics; it is about upholding the fundamental dignity and rights of every citizen of Nepal.

## Recommendations for a strengthened and resilient system

To address this crisis comprehensively, I argue that Nepal must move beyond isolated interventions and adopt a multi-pronged, systemic approach grounded in international best practices and adapted to the local context. The solution to the challenge of substandard medicines is complex, requiring a foundational shift from reactive detection to proactive prevention and quality assurance throughout the entire supply chain.

First, the cornerstone of any effective strategy must be the robust implementation of a national quality assurance framework based on established WHO principles (19). This involves the mandatory adoption and enforcement of Good Manufacturing Practices (GMP) for domestic producers, ensuring that quality is built into products from the outset (20). Critically, this must be complemented by the nationwide implementation of Good Storage Practices (GSP) and Good Distribution Practices (GDP), which are presently lacking in Nepal (2). As detailed in WHO guidelines, this requires establishing a comprehensive Quality Management System that governs every stage of the supply chain, from procurement to delivery. This includes temperature-controlled storage and transport, proper documentation to ensure traceability, and rigorous training for all personnel involved (21, 22). The goal is to create a secure, transparent, and controlled environment where opportunities for degradation or the introduction of falsified products are minimized.

Second, this preventative framework must be supported by strengthening practices at the point of care through the enforcement of Good Pharmacy Practices (GPP). While pharmacist-led detection of visibly substandard medicines is challenging and should not be the primary line of defense, pharmacists are integral to maintaining quality assurance (23). The US Agency for International Development (USAID) funded Medicines, Technologies, and Pharmaceutical Services (MTaPS) program has already highlighted the significant impact of GPP implementation on product quality and patient safety in Nepal (24). The urgency of this is underscored by recent local evidence from the Supervision, Performance Assessment, and Recognition Strategy (SPARS) pilot. The baseline assessment from the SPARS pilot, conducted in 2022 across 284 health facilities, revealed alarmingly poor medicines management practices, with an overall median score of only 34% across key performance indicators. Particularly low scores in domains such as storage management and stock management highlight critical, systemic gaps that a robust GPP framework is precisely designed to address (25). Therefore, a renewed focus on GPP—including proper storage, inventory management using the First-Expired-First-Out (FEFO) principle, and patient counseling (26, 27)—is not merely a recommendation but an evidence-based necessity to safeguard medicine quality at the final stage of the supply chain.

Third, a fundamental overhaul of the national regulatory body is imperative. While establishing a new, fully autonomous commission with civil society oversight represents an ideal long-term goal, a more pragmatic and immediate strategy is to empower the existing Department of Drug Administration (DDA). Given Nepal's resource constraints, strengthening the current regulatory infrastructure is a more feasible first step. Following successful models from other nations, such as the autonomous South African Health Products Regulatory Authority (SAHPRA) (28), granting the DDA greater operational and financial autonomy would be transformative. An empowered DDA could then engage in the kind of international collaboration essential for modern pharmacovigilance, such as the recent Memorandum of Understanding between SAHPRA and Australia's Therapeutic Goods Administration (TGA) to share regulatory information and expertise (29). Recent progress within Nepal indicates a readiness for such advancement; for instance, with support from USAID MTaPS, the DDA has begun modernizing its document management system by installing secure, access-controlled repositories to increase efficiency and security (30). While these foundational improvements are commendable, they must be viewed as the first steps in a much longer journey. Full regulatory maturity requires sustained investment to build upon these gains. This must include a significant increase in the number of trained inspectors and a shift toward a risk-based inspection model that prioritizes systematic laboratory testing of products from importers and wholesalers before they enter the market, thereby ensuring quality at the source rather than attempting to recall products that have already been dispensed.

Finally, these systemic and regulatory reforms should be amplified by leveraging modern technology and strengthening post-market surveillance. A vital component underpinning all these reforms is compliance with Good Practice (GxP) principles. GxP is an acronym for a collection of quality guidelines and regulations designed to ensure that products within heavily regulated industries, such as pharmaceuticals, are consistently safe, effective, and fit for their intended use (31). This framework is not a single standard but an ecosystem of practices—including GMP, GSP, and GPP—that collectively safeguard product integrity. While technology is not a panacea, rigorous track-and-trace systems—using technologies like blockchain or simple 2D barcoding—offer a powerful tool for enforcing GxP compliance and achieving end-to-end visibility. This enables regulators to verify authenticity and rapidly identify diversions in the supply chain (32, 33). Such systems, proven to be cost-effective in settings like Bangladesh (34), would support the broader GxP framework (31) and provide crucial data for the empowered DDA. Within this enhanced surveillance system, pharmacists, supported by legally protected reporting mechanisms, become vital nodes for reporting suspected adverse events or quality issues, thereby creating a feedback loop that strengthens the entire regulatory ecosystem (35).

## Conclusion

The integrity of a nation's medicine supply is a direct reflection of its commitment to health equity and social justice. For Nepal, the continued presence of substandard medicines in the supply chain represents a fundamental contradiction to its goal of achieving universal health coverage. The systemic reforms proposed—implementing a robust GxP framework, empowering an autonomous regulator, and leveraging technology for surveillance—are not merely technical exercises; they are essential acts of building trust with the nation's most vulnerable citizens and affirming their right to safe, effective healthcare. Moving forward, the challenge is not one of awareness, but of political will and sustained action. Ensuring the quality of every pill and vial is not just a matter of public health policy—it is a fundamental test of Nepal's promise of equitable healthcare for all.
